# OTME-15. Physical Confinement Induces Diverse Transcriptomic Changes and Chemoresistance in Migrating Glioblastoma Cells

**DOI:** 10.1093/noajnl/vdab070.066

**Published:** 2021-07-05

**Authors:** James Battiste, Anish Babu, Rachel Sharp, Sydney Scott, Ian Dunn, Chad Glenn, Young-tae Kim, Kenneth Jones

**Affiliations:** 1 OU Health Science Center, Oklahoma City, OK, USA; 2 Department of Neurosurgery, Oklahoma City, OK, USA; 3 Stephenson Cancer Center, Oklahoma City, OK, USA; 4 Department of Cell Biology, Oklahoma City, OK, USA; 5 UT Arlington, Arlington, TX, USA

## Abstract

Glioblastoma multiforme (GBM) cells migrating in physically confined environments are affected by mechanical stress that potentially lead to transcriptomic changes. To simulate those stresses, microfluidic channels were made with micro-patterned polydimethylsiloxane (PDMS) replicating the physical microenvironment of white matter tracts by confining the cells in linear channels similar to the space between axons. We employed a combination of microarray transcriptomic profiling and single cell-sequencing analyses to investigate cells undergoing linear confined space migration (LCSM). GBM cells spontaneously migrate through confined spaces along 5x5 mm (height/width) microfluidic channels, 0.5 to 5 mm in length. Our previous studies demonstrated that cells migrating in LCSM are more resistant to treatment with temozolomide than the same cells growing in standard monolayer culture (SMC). Cells in confined migration evaluated by microarray-based transcriptomic profiling demonstrated that linear confined migration induces increased expression in pathways involving angiogenesis, cell adhesion, cell motility, DNA damage repair, extracellular matrix structure, HIF1α, and others. Single cell transcriptomic analysis could identify GBM cells in different migratory states (LCSM vs. SMC), and similar pathways were seen upregulated with additional changes in cholesterol biosynthesis pathways and cell cycle regulation pathways. Trajectory Inference aligned single cells according to changes in migration status and demonstrated transcript changes during LCSM were progressive but generally reversible on return to SMC. Pathway analyses showed alterations in the cholesterol biosynthesis pathway and cell cycle regulation in cell clusters of confined migrating cells. Molecular studies confirmed that cholesterol biosynthesis pathway regulatory genes (SQLE, MVD, and HMGCR) are upregulated during LCSM. Expression analysis demonstrated increased G1 phase delay in confined migrating cells (LCSM) confirmed by FUCCI expression analysis. We propose that migration in linear confined spaces like white matter structures produces significant transcriptome changes that produce chemoresistance as a new mechanism for treatment resistance of Glioblastoma.

